# Unlocking the molecular realm: advanced approaches for identifying clinically and environmentally relevant bacteria

**DOI:** 10.1590/1414-431X2023e12894

**Published:** 2023-10-13

**Authors:** M.R.F. da Silva, K. Souza, T. Bezerra, T. Silva, D. Fernades, F. Silva, L. Araújo, A. Almeida, M. Oliveira

**Affiliations:** 1 Departamento de Bioquímica, Universidade Federal de Pernambuco, Recife, PE, Brasil; 2 Departamento de Microbiologia, Instituto Aggeu Magalhães, FIOCRUZ PE, Recife, PE, Brasil; 3 Departamento de Tecnologia Bioquímico-Farmacêutica, Universidade de São Paulo, São Paulo, SP, Brasil

**Keywords:** Bacterial pathogens, Clinical and environmental samples, Diagnosis, Molecular methods

## Abstract

Rapid, effective, and specific identification of clinical and environmental bacterial pathogens is of major importance for their control. Traditionally, bacteria have been identified by phenotypic methods based on morphological, biochemical, and metabolic properties. While these methods are very useful in clinical practice, they have limitations including a poor ability to differentiate within and between species and time-consuming workflows. Newly developed molecular methods can greatly improve the accuracy of taxonomic characterization, identifying specific strains of medical or environmental importance. However, due to high costs and the need for trained professionals, these methods are not yet routine in diagnostic laboratories. Thus, disseminating knowledge on advances in molecular identification techniques is pivotal to make these methodologies accessible. The objective of this work was to review and discuss current molecular techniques for bacteria identification aiming to track and monitor microbial agents in clinical and environmental samples.

## Introduction

Bacteria are microorganisms with an extraordinary ability to adapt to different environments, and many of them are etiological agents of a wide range of diseases, especially those resulting from the ingestion of contaminated food and water. During their evolutionary process, bacteria have developed several mechanisms to survive and grow in unfavorable environments. One of these mechanisms is horizontal gene transfer (HGT), which contributes to increasing resistance and making treatment and infection control more difficult (1,2). Therefore, a quick, effective, and specific diagnostic method is crucial in the control of the dissemination of bacteria. Traditionally, bacteria have been identified by a panel of phenotypic tests, including morphology, Gram staining, and metabolism of biomolecules (e.g., citric acid, urea, sulfur compounds, and others). These methods provide the most probable, but not the definitive, identification of the microorganism. However, intra- and inter-species variations in these phenotypic features can lead to misleading test results (3,4). In this context, the use of molecular methods represents an improvement in the clinical diagnosis of bacterial infections and allows for faster and more accurate taxonomic characterization in clinical and environmental microbiological diagnoses (5). In view of this, the objective of this work was to carry out an analysis of molecular techniques, which help in the identification of microbial agents present in clinical and environmental samples, contributing to the proposed therapies employed and helping hospital and environmental infection control bodies.

## Material and Methods

**Figure 1:**
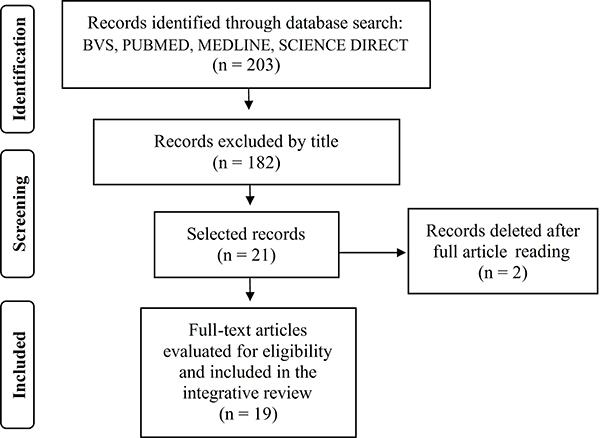
Inclusion and exclusion flowchart of selected articles according to the Preferred Reporting Items for Systematic Reviews and Meta-Analyses (PRISMA) protocol guidelines.

This study is an integrative literature review aiming to inform about alternative molecular methods for the identification of bacteria of clinical and environmental origin. Our inclusion criteria were as follows: full texts compatible with the subject of interest and indexed in international databases. Duplicate publications, bibliographic reviews, editorials, dissertations, and theses were excluded. The research was conducted in the PubMed, Medline, Science Direct, and BVS databases using descriptors from the Descriptors in Health Sciences (DeCS) platform (https://decs.bvsalud.org/), including Molecular Diagnosis Techniques, Bacteria, Polymerase Chain Reaction (PCR), Bacterial Infections, and Environmental Change. The complete strategy for data collection, storage, and analysis is presented in Figure 1. Using the established descriptors and inclusion criteria, this integrative review found 203 articles, of which 19 were selected. Of these, eight (43%) were identified in PubMed, seven (36%) in Science Direct, two (10%) in BVS, and two (10%) in Medline.

## Results and Discussion

The results allowed the identification of the main molecular techniques used for the detection of clinically and environmentally important pathogens, which were categorized into three main topics: detection of clinical pathogens, detection of environmental pathogens, and detection of clinical and environmental pathogens. Supplementary Table S1 summarizes the results, showing the field and approach, title, objective, molecular technique used, authorship, and year of the study.

### Detection of clinical pathogens

Microbial infections have increased over the years, particularly those caused by antibiotic-resistant microorganisms, posing a threat to humans due to their high mortality rates. Molecular techniques for etiological diagnosis of infections allow the detection of specific genes and are faster and more sensitive than traditional culture-based approaches.

A prominent molecular technique for bacterial identification is the Loop-Mediated Isothermal DNA Amplification Assay (LAMP) developed by Notomi et al. (6), which is simple, fast, and inexpensive. It amplifies nucleic acid in a more sensitive, simple, and efficient manner than conventional PCR. The amplification occurs at a constant temperature using the Bst DNA polymerase enzyme (*Bacillus stearothermophilus* DNA polymerase protein with 5'→3' polymerase activity) and a set of four to six primers specifically designed to amplify six to eight regions in the target gene. The amplification can be performed in a water bath, and the results can be directly visualized without the need for equipment, as well as through the use of chromogenic dyes for amplification detection (7). The LAMP technique has also been described as more sensitive than conventional PCR for detecting vancomycin resistance genes in *Enterococcus faecalis* and *E. faecium* directly in patients' urine, successfully differentiating isolates of these two species (8). LAMP has also been recognized as a promising diagnostic tool for more sensitive and effective detection of virulence-associated genes in *Vibrio cholera* (9).

The cross-priming amplification (CPA) assay has been shown to be an easy, fast, specific, and sensitive molecular method, especially when coupled with other techniques such as the nanoparticle-based lateral flow biosensor (CPA-LFB) for detecting bacteria and other microorganisms. As an isothermal amplification method, the CPA reaction occurs at a constant temperature ranging from 58 to 69°C in approximately 40 min, and amplification reaction products can be visibly detected by an LFB without the need for special equipment. The method is based on the binding of antibodies (embedded in the LFB) and specifically labeled haptens on the 5' end of the primers. CPA-LFB has been reported as a potential diagnostic tool for *Mycoplasma pneumoniae* in clinical laboratories. Moreover, this method allows the visual and rapid detection of *Candida albicans* fungus in clinical samples, showing concordant results with gold standard culture methods or real-time polymerase chain reaction (RT-qPCR).

The multi-criteria decision analysis (MCDA) assay is experimentally similar to the LAMP assay, but the MCDA technique has a higher sensitivity for bacterial detection compared to LAMP (10,11). Thus, MCDA becomes an easy, fast, specific, and very sensitive molecular method for detecting microorganisms, especially when combined with and/or adapted to other techniques. Other methods for pathogen identification also deserve attention, including semi-automated qPCR systems, such as the GeneXpert tests from Cepheid (for pathogen detection and antimicrobial resistant genes), ribosomal genetic sequencing, and MALDI-TOF (matrix-assisted laser desorption ionization time of flight mass spectrometry) (12,13).

### Detection of environmental pathogens

The increasing exploitation of natural resources also requires knowledge of potential sources of pollution and their impact on the environment. Due to anthropogenic activities affecting water, soil, and air quality, it is imperative that strategies be developed to identify and determine potential sources of pollution and the organisms affected. Many studies have been conducted in various ecosystems to identify potentially harmful pathogens to the environment and human health. The traditional diagnostic methods are not keeping pace with the rapid spread of environmental pathogens, as they are culture-based, often time-consuming, and require additional biochemical testing for confirmation (13).

The use of molecular techniques such as RT-qPCR technology is growing at the expense of traditional techniques and becoming one of the most frequently used methodologies for environmental studies. RT-qPCR has high sensitivity and broad applicability to a variety of pathogens and sample types, making it a viable and rapid technique (14,15). In epidemiological studies, the *Streptococcus agalactiae* species was identified by RT-qPCR from brain tissue of dead fish, thus elucidating the causative agent of the high mortality in Nile tilapia (*Oreochromis niloticus*) (16). RT-PCR technology is also useful for monitoring recirculating system programs to assess water quality and animal health status. RT-PCR analyses of environmental samples such as water, sludge, or tank debris and clinically diseased fish have allowed the distinction that *Pseudoloma neurophilia* or *Myxidium streisingeri* were found exclusively or predominantly in fish, whereas mycobacteria were predominantly present in environmental samples. Therefore, the combination of fish and environmental sampling appears to be necessary for the detection of a wide range of infectious agents in zebrafish colonies using RT-PCR technology (17). RT-qPCR also allows the identification of microorganisms other than bacteria with a rapid response time and high sensitivity and specificity.

In aquaculture, detection of pathogens in the early stages of infection is crucial for disease control. A RT-qPCR procedure using the SYBR Green I dye was useful for quantitatively identifying Tilapia Lake Virus (TiLV) in tilapia (*O. niloticus*) tissues. This approach enabled quantification of TiLV down to 2 copies and therefore proved to be exceptionally useful for diagnosing TiLV in subclinical cases. Some advantages of this technique include its quantitative nature, high sensitivity, high specificity, and timely results, making it a valuable tool for establishing rapid disease control strategies in aquaculture (18).

The LAMP technique has been widely used for various purposes in the identification of pathogens, whether of clinical or environmental importance. Under minimal buffering conditions, after DNA amplification, the technique produces hydrogen ions that lower the pH of the reaction. This characteristic has been applied to visually detect amplified DNA of *Escherichia coli* using xylene orange, a pH-dependent dye (19). The versatility of the LAMP assay also allows for its combination with a lateral flow dipstick (LFD), which, in addition to high specificity and sensitivity, offers the advantage of requiring less time than common molecular techniques such as conventional PCR for the detection of *Vibrio parahaemolyticus* from pure cultures or experimentally contaminated food samples (20). A portable LAMP device coupled with a syringe filter-based DNA extraction method enabled the rapid detection of fecal indicator bacteria (*E. coli* and *E. faecalis*) for microbial water quality assessment without requiring standard laboratory equipment or specialized training for these analyses (21).

A DNA-based colorimetric magnetogenosensor coupled to LAMP (LAMP biosensor assay) provides simple and rapid visual detection of pathogenic *Leptospira*, the causative agent of leptospirosis. The biosensor operates through a DNA hybridization system in which a specifically designed probe captures the target LAMP amplicons (22).

MCDAs have also been utilized in environmental samples, such as the MCDA-LFB assay based on multiple cross displacement amplification and nanoparticle-based LFB, demonstrating excellent specificity and sensitivity for the rapid detection of *L. interrogans* in human, animal, and environmental samples. The assay was completed in 70 min and showed faster results compared to PCR methodology, as well as high specificity and sensitivity, with all pathogenic *L. interrogans* isolates testing positive and all non-Leptospira and non-pathogenic samples testing negative (23).

Several rapid methodologies have been developed for detection and quantification of pathogens based on specific genes and proteins. In addition to being fast, they also provide improved detection potential and specificity. The ability to quantify pathogens has proven useful as a prognostic indicator and for monitoring treatment response in many infections. Digital droplet PCR (DD-PCR) is an enhanced PCR technology that can clonally amplify and quantify DNA and RNA. DD-PCR is more sensitive than conventional PCR as it detects low concentrations of DNA and can be applied in pathogen detection, gene mutation, gene copy number variation, mRNA expression level, and DNA modification (24). DD-PCR is particularly useful for detecting genetically modified organisms (GMOs) and pathogens in food, such as *Salmonella* spp., which is one of the most important pathogens and the leading cause of foodborne diseases (25,26). The availability of various commercial platforms opens up many opportunities for the use of DD-PCR in clinical microbiology laboratories (27,28).

The rapid viability polymerase chain reaction (RV-PCR) method uses a shift in the cycle threshold after incubation to confirm the presence of viable organisms. It is capable of detecting and identifying viable cells of *Francisella tularensis* in environmental samples at least twice as fast as the current plate culture-based method, while being lightweight, generating fewer residues, and requiring minimal labor. Due to its historical use as a biological weapon and the occurrence of natural tularemia outbreaks, there is a need for rapid and sensitive analytical methods for the detection of viable *F. tularensis* in environmental samples. The RV-PCR method can help improve laboratory capacity for rapid, reliable, and high throughput analysis of samples to effectively respond to an intentional, accidental, or natural incident resulting in *F. tularensis* contamination of water infrastructure (29). This procedure also allows rapid detection of viable *Clostridioides difficile* spores in the environment during epidemiological investigations and can potentially be used to determine if cleaning methods are adequate for disinfecting *C. difficile* spores (30). RV-PCR can greatly reduce the time required to detect viable *C. difficile* spores as sample incubation is only necessary for germination (22 h or less) instead of colony formation, which can take up to 7 days. Additionally, RV-PCR can quickly confirm or deny the organism's identity while confirming viability.

Another molecular technique used for analyzing environmental samples is viability-based quantitative PCR (vPCR) with propidium monoazide (PMA). The PMA dye irreversibly intercalates with extracellular DNA or DNA in cells with compromised membranes, thereby preventing amplification by qPCR and reducing the qPCR signal from DNA originating from non-viable sources. Studies on viability-based quantification of antibiotic resistance genes (ARGs) and human fecal markers in wastewater demonstrated that ARGs and other target genes were significantly lower in the viable cell fraction of effluent samples compared to total gene concentrations (31).

### Detection of clinical and environmental pathogens

Molecular techniques are widely used for pathogen characterization in body fluids, and their use for bacterial analysis in environmental samples is increasing. The repetitive element palindromic PCR (rep-PCR) technique utilizes complementary primers flanking repetitive regions to amplify DNA fragments of multiple sizes depending on the number of repetitive sequences found in bacterial genomes, making it useful for clinical or environmental analyses (32). Therefore, the technique has the advantage of using purified genomic DNA, crude bacterial cell lysates, or infected samples to generate DNA fingerprint profiles. Additionally, rep-PCR products of different sizes can be visualized through ethidium bromide-stained gels, computer-assisted detection and data storage methods, or laser scanning of fluorophore-labeled amplification products. DNA fingerprint patterns can be compared to estimate relative degrees of similarity between isolates and determine if the isolates are clonally related. The use of rep-PCR and next-generation sequencing (NGS) provided molecular information that demonstrated clonality and identified potential environmental sources of contamination, helping frontline infection control teams develop control strategies against *S. marcescens* outbreaks in a neonatal intensive care unit (33).

Another promising technique is recombinase polymerase amplification (RPA), which is highly specific and can be performed at normal temperatures. Amplifications can be visualized using lateral flow strip (LFS) assays or real-time fluorescence, offering advantages in detection flexibility and utility in endemic environments (34). RPA combined with LFD has been used to detect *V. alginolyticus*, a pathogen responsible for significant economic losses in marine culture. The test was based on the detection of the toxR virulence gene and showed high specificity for pathogenic strains of *V. alginolyticus* without any cross-reactivity with other *Vibrio* species or pathogenic bacteria (35). RPA combined with LFD also enabled rapid visual detection of *V. parahaemolyticus* with high specificity within 25 min at temperatures ranging from 35 to 45°C (36).

Point-of-care (POC) systems have proven to be advantageous by providing rapid response time and timely decision making in patient management. The U.S. Food and Drug Administration (FDA) has released several POC molecular platforms or Clinical Laboratory Improvement Amendments (CLIA), which exhibit sensitivity and specificity similar to nucleic acid amplification tests (NAAT) (37).

The performance of the Roche cobas Liat^®^ platform and the Roche cobas Liat^®^ Group A *Streptococcus* (GAS) assay were compared to routine real-time PCR in two retail-based convenience clinics by Donato et al. (38). The authors assessed the instrument's accuracy and failure rate and monitored environmental contamination when the test was performed by minimally trained end users (38). The cobas Liat (Lab-in-a-tube) platform and the GAS assay demonstrated reliable performance in the end user setting. Therefore, it can serve as a rapid POC option for routine diagnostic testing for certain infectious diseases, including GAS infections. The Roche cobas Liat platform also showed a rapid response for the identification of *Streptococcus pyogenes* compared to rapid antigen detection test (RADT). The Roche cobas Liat is a compact, automated platform that is easy to handle and has been used for the detection of various bacterial and viral pathogens, such as Strep A, influenza A/B, respiratory syncytial virus (RSV), SARS-CoV-2, and the detection of the toxigenic B toxin gene (TCD B) of *Clostridium difficile* (39).

**Figure 2:**
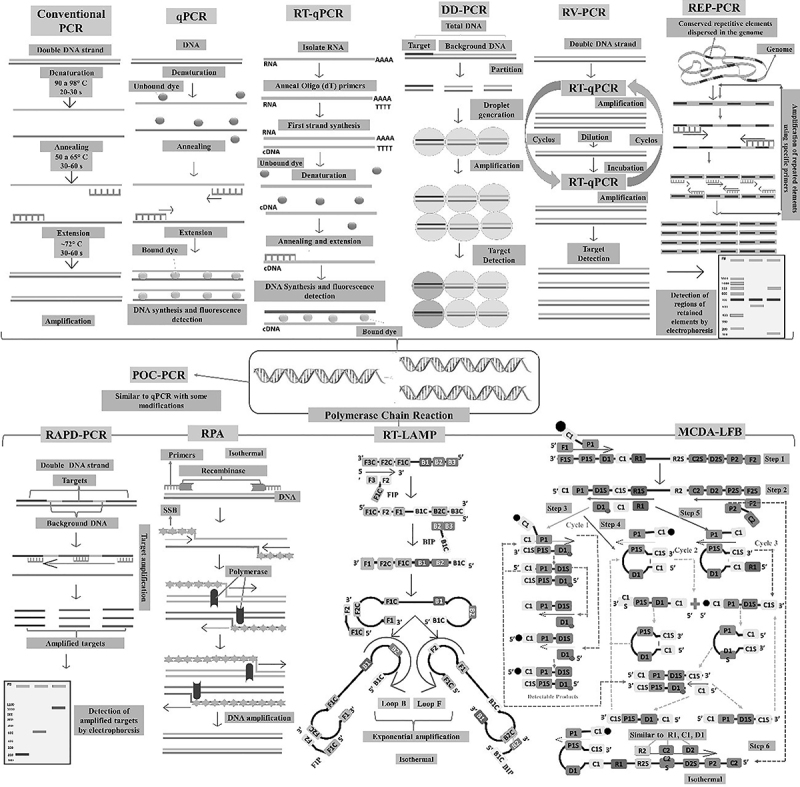
Schematic representation of the main molecular techniques used to identify pathogens of clinical and environmental importance. PCR: polymerase chain reaction; RT-qPCR: real-time PCR; DD-PCR: digital droplet PCR; RV-PCR: rapid viability PCR; REP-PCR: repetitive element palindromic PCR; POC-PCR: point-of-care; RAPD-PCR: randomly amplified polymorphic DNA PCR; RPA: recombinase polymerase amplification; RT-LAMP: real-time loop-mediated isothermal DNA amplification assay; MCDA-LFB: multi-criteria decision analysis lateral flow biosensor.

In summary, numerous techniques can be used to identify microorganisms of clinical and environmental importance, and the techniques addressed in this review are shown in Figure 2.

## Conclusion

The development of molecular techniques has revolutionized diagnostic methodology, contributing effectively to the identification of microorganisms and their different strains, as well as to the differentiation between pathogenic and non-pathogenic organisms, and the analysis of the geographical and temporal distribution of pathogens of clinical and environmental importance. Conventional PCR has been improved and opened new diagnostic possibilities by providing more specific, accurate, and faster methods for molecular identification of microorganisms. Some molecular techniques such as LAMP, MCDA-LFB, RPA-LFD, and the Roche cobas Liat platform are becoming more widely known and provide positive and efficient results due to their easiness and rapidity and higher sensitivity, specificity, and reproducibility. However, some of them remain unknown to most diagnostic laboratories and researchers, and if known, they are not accessible due to the equipment cost, the need for trained professionals, and constant updates. Thus, advances in molecular identification techniques and dissemination of knowledge are necessary to make these methodologies accessible and help improve personalized treatments and update epidemiological surveillance agencies. Studies in this area are very promising and full of innovative possibilities.

## Supplementary Material

Click to view [pdf].
