# **Erratum notice for:** “Unlocking the molecular realm: advanced approaches for identifying clinically and environmentally relevant bacteria” [Braz J Med Biol Res 2023: 56, e12894]

**DOI:** 10.1590/1414-431X2024e12894erratum

**Published:** 2024-12-02

M.R.F. da Silva^1^ https://orcid.org/0000-0003-0203-4506, K. Souza^1^ https://orcid.org/0000-0003-2627-7385, T. Bezerra^2^ https://orcid.org/0000-0002-9759-1710, T. Silva^1^ https://orcid.org/0000-0002-8142-1995, D. Fernades^2^ https://orcid.org/0000-0002-5062-8248, F. Silva^1^ https://orcid.org/0000-0003-1264-5597, L. Araújo^1^ https://orcid.org/0000-0002-5537-6641, A. Almeida^2^ https://orcid.org/0000-0002-8304-3182, and M. Oliveira^1^ https://orcid.org/0000-0001-5188-3243

^1^Departamento de Bioquímica, Universidade Federal de Pernambuco, Recife, PE, Brasil

^2^Departamento de Microbiologia, Instituto Aggeu Magalhães,, FIOCRUZ PE, Recife, PE, Brasil

Correspondence: M.R.F. da Silva: <milena.freire@ufpe.br>

Erratum for: Braz J Med Biol Res | doi: 10.1590/1414-431X2023e12894 | PMID: 37851790 | PMCID: PMC10578128

The correct affiliation of the author F. Silva is ^1^Departamento de Bioquímica, Universidade Federal de Pernambuco, Recife, PE, Brasil where this research was carried out. The institution ^3^Departamento de Tecnologia Bioquímico-Farmacêutica, Universidade de São Paulo, São Paulo, SP, Brasil has been deleted.

